# Accuracy of pancreatic stone protein for the diagnosis of infection in hospitalized adults: a systematic review and individual patient level meta-analysis

**DOI:** 10.1186/s13054-021-03609-2

**Published:** 2021-05-28

**Authors:** Josef Prazak, Irina Irincheeva, Martin J. Llewelyn, Daiana Stolz, Luis García de Guadiana Romualdo, Rolf Graf, Theresia Reding, Holger J. Klein, Philippe Eggimann, Yok-Ai Que

**Affiliations:** 1 Department of Intensive Care Medicine, INO E-403, Inselspital, Bern University Hospital, University of Bern, 3010 Bern, Switzerland; 2 CTU Bern, University of Bern, Bern, Switzerland; 3 Brighton and Sussex Medical School, Falmer, BN1 9PS UK; 4 Clinic of Pulmonary Medicine and Respiratory Cell Research, University Hospital Basel, Basel, Switzerland; 5 Biochemistry Department, Santa Lucia University Hospital, Cartagena, Spain; 6 Department of Visceral and Transplantation Surgery, Universitätsspital Zürich, Zurich, Switzerland; 7 Department of Plastic Surgery and Hand Surgery, Burn Center Zurich, Universitässpital Zürich, Zurich, Switzerland; 8 Department of Locomotor Apparatus, Lausanne University Hospital (CHUV) and University of Lausanne, Lausanne, Switzerland

**Keywords:** Pancreatic stone protein, PSP, Infection, Biomarker

## Abstract

**Background:**

Accurate biomarkers to diagnose infection are lacking. Studies reported good performance of pancreatic stone protein (PSP) to detect infection. The objective of the study was to determine the performance of PSP in diagnosing infection across hospitalized patients and calculate a threshold value for that purpose.

**Methods:**

A systematic search across Cochrane Central Register of Controlled Trials and MEDLINE databases (1966–March 2019) for studies on PSP published in English using ‘pancreatic stone protein’, ‘PSP’, ‘regenerative protein’, ‘lithostatin’ combined with ‘infection’ and ‘sepsis’ found 44 records. The search was restricted to the five trials that evaluated PSP for the initial detection of infection in hospitalized adults. Individual patient data were obtained from the investigators of all eligible trials. Data quality and validity was assessed according to PRISMA guidelines. We choose a fixed-effect model to calculate the PSP cut-off value that best discriminates infected from non-infected patients.

**Results:**

Infection was confirmed in 371 of 631 patients. The median (IQR) PSP value of infected versus uninfected patients was 81.5 (30.0–237.5) versus 19.2 (12.6–33.57) ng/ml, compared to 150 (82.70–229.55) versus 58.25 (15.85–120) mg/l for C-reactive protein (CRP) and 0.9 (0.29–4.4) versus 0.15 (0.08–0.5) ng/ml for procalcitonin (PCT). Using a PSP cut-off of 44.18 ng/ml, the ROC AUC to detect infection was 0.81 (0.78–0.85) with a sensitivity of 0.66 (0.61–0.71), specificity of 0.83 (0.78–0.88), PPV of 0.85 (0.81–0.89) and NPV of 0.63 (0.58–0.68). When a model combining PSP and CRP was used, the ROC AUC improved to 0.90 (0.87–0.92) with higher sensitivity 0.81 (0.77–0.85) and specificity 0.84 (0.79–0.90) for discriminating infection from non-infection. Adding PCT did not improve the performance further.

**Conclusions:**

PSP is a promising biomarker to diagnose infections in hospitalized patients. Using a cut-off value of 44.18 ng/ml, PSP performs better than CRP or PCT across the considered studies. The combination of PSP with CRP further enhances its accuracy.

**Supplementary Information:**

The online version contains supplementary material available at 10.1186/s13054-021-03609-2.

## Background

Severe infections are leading causes of morbidity and mortality among hospitalized patients [1, 2]. Early detection of life-threatening infection is crucial to improving outcomes [1]. To date, none of the circulating blood biomarkers or signatures of the immune response that have been investigated [3, 4] detect life-threatening infection quickly enough and with an acceptable certainty [5]. Two markers [C-reactive protein (CRP) and procalcitonin (PCT)] are widely used [6–10] despite their sub-optimal performance [11].

Pancreatic stone protein (PSP) is a pro-inflammatory mediator that binds to polymorphonuclear cells and triggers their activation in vitro (reviewed in [12]); it is a recently described biomarker of infection whose performance has been thoroughly evaluated in several patient populations and in different clinical settings, including emergency rooms (ERs) [13] and intensive care units (ICUs) [14–18]. PSP was able not only to diagnose infection [14, 15, 18] but also to characterize its severity [16, 17] as well as to predict its outcome [16, 17, 19]. Nevertheless, a clinically useful PSP threshold level has not yet been defined.

We aimed to perform an individual patient level meta-analysis of published data to determine the performance of PSP in detecting infection, to propose a threshold value for that purpose and to validate it across heterogeneous populations.

## Methods

### Search strategy and selection criteria

A systematic literature search was performed based on the following search strategy prepared according to PRISMA individual patient data guidelines (Additional file 1: Tables S1 and S2) [20]. The databases searched were the Cochrane Central Register of Controlled Trials (CENTRAL) and MEDLINE (1966–March 2019). The search was restricted to original human clinical trials on PSP/reg published in English before March 2019 that assessed the performance of PSP for the initial detection of infection in hospitalized adults (see definition of infection for each study in Additional file 1: Table S3). We used ‘pancreatic stone protein’, ‘PSP’, ‘regenerative protein’, ‘infection’, ‘sepsis’ and ‘lithostatin’ as keywords. Paediatric trials and autopsy studies were excluded.

**Fig. 1:**
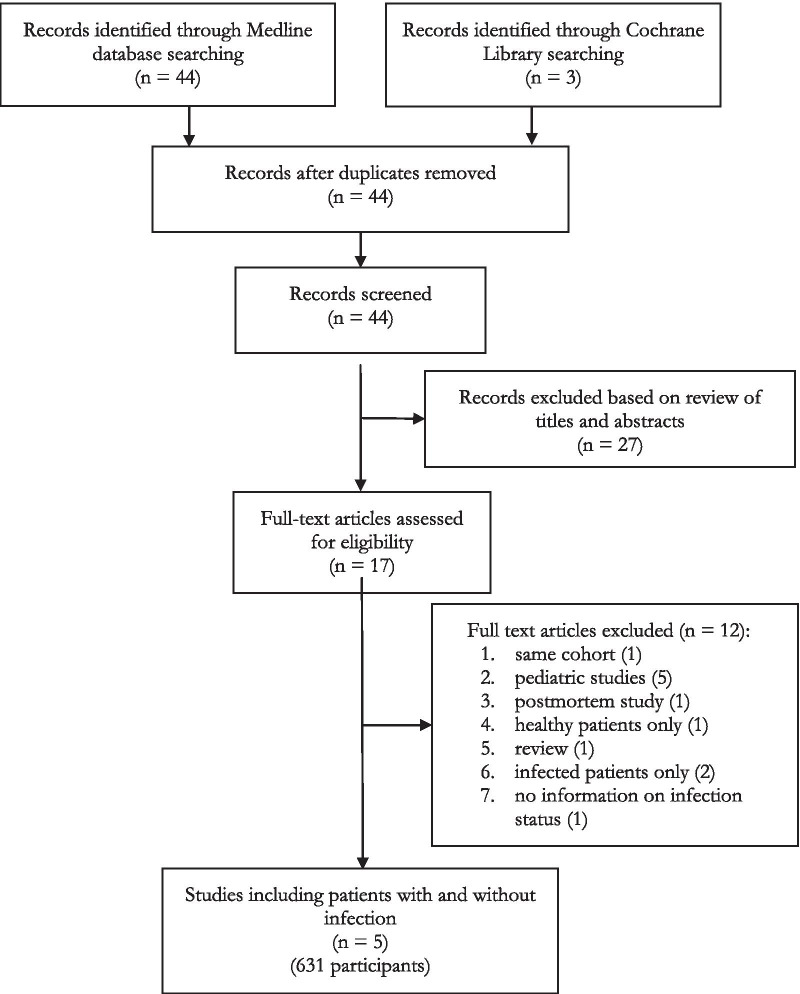
Study selection

Two reviewers (JP and YAQ) independently assessed trial eligibility based on titles, abstracts, full-text reports and further information from investigators as needed (Fig. 1). Study protocols and unedited databases containing anonymized individual patient data were obtained from investigators of all eligible trials.

The Cantonal Ethical Committee of the State of Bern (#2018-01356) reviewed and approved the protocol of this systematic review and meta-analysis in 2018 (see Additional file 2: Appendix) and the respective ethical committees approved all specific studies.

### Assessment of data validity

All raw data were received with patient-specific anonymized identification and included at least the following information: age, sex, confirmed infection/non-infection and values of PSP, CRP and PCT. For Klein et al*.,* we did not receive PCT values or gender data. Death outcomes and days to death were described only in Keel et al*.* and Llewelyn et al. Data from each trial were first checked for duplicates and then against reported results. Queries were resolved with the principal investigator, trial data manager or statistician when needed. Cases with missing PSP values were excluded. In all eligible subjects, PSP levels were measured according to the same protocols and techniques developed by Rolf Graf’s team at the Universitätsspital Zürich, Switzerland. Llewelyn et al*.* used plasma instead of serum; however, we did not observe any considerable variations in main PSP values between plasma and serum. Biomarker data were checked for consistency and variability within and between studies to detect possible bias using known ranges, boxplots and estimated densities of log-transformed values (Additional file 3: Figures S1 and S2). PCT exhibits increased within- and between-study variability (higher interquartile range and much higher outlier points) while compared to PSP and CRP.

### Data analysis

We included all adult patients admitted to either an ER or an ICU and in whom PSP was measured to diagnose infection. For both studies with multiple time points, we restricted the analysis on day 5 after severe trauma for Keel et al*.* and on day 2 after cardiac surgery for Klein et al. The primary objective was to calculate the optimal PSP threshold value to discriminate infected from non-infected patients. The secondary objectives were: (1) to compare the PSP accuracy, its negative and positive predicting values as well as its positive and negative likelihood ratio to those of PCT and CRP for detecting infected patients, and finally (2) to explore the value of models using different combinations of the three biomarkers to further improve the detection of infection.

Because data exploration showed skewness (Additional file 3: Figures S1 and S2) in most continuous variables, these were reported as medians and interquartile ranges (IQRs). Categorical variables were reported as frequencies and percentages.

### Statistical analysis

#### Meta-analysis model selection and heterogeneity assessment

We performed an individual patient data meta-analysis and evaluated three models according to the approach proposed by Steyerberg et al. [21] (Additional file 1: Table S4 and Additional file 4: Supplemental Methods). A ‘fully stratified’ random-effect model (random intercept and random PSP effect) was compared to a mixed-effects model with random study effect but fixed treatment effect, and a fixed-effect model (Additional file 1: Tables S4 and S5). Even though the fully stratified and the mixed-effects model provided better ROC AUCs (Additional file 1: Tables S7 and S9), we selected the fixed-effect model as it was the only one among the three models considered that provided a unique PSP threshold (Additional file 1: Tables S6, S7 and S9).

#### Single biomarker approach

ROC curves were constructed for all untransformed biomarkers of interest (PSP, PCT and CRP) by varying the cut-off values distinguishing infection from non-infection. The best PSP, PCT and CRP cut-off values to detect infected patients were determined using the Youden’s index applied to their respective ROC curves. The cut-off specific sensitivity, specificity, positive predictive value (PPV), negative predictive value (NPV), positive (PLR) and negative (NLR) likelihood ratio were reported for PSP and further compared to those of PCT and CRP.

#### Approach combining biomarkers

To explore the potential added value of a model combining the biomarkers to detect infection, we fitted to the data three logistic regression models with biomarkers as continuous covariates and tested the following combinations of biomarkers: PSP and CRP, PSP and PCT; PSP, CRP and PCT.

#### Statistical packages and version

All *P* values were two-sided, and statistical significance was set at a *P* value of less than 0.05. All analyses were performed using R for Windows (version 3.0.1) with the following packages: ROCit, reportROC, pROC and OptimalCutpoints packages [22–24].

## Results

### Study and data collection

Among the 44 records published before March 2019 and identified through the literature search, 17 full-texts were further assessed for eligibility. Twenty-seven records were excluded based on review of title and abstracts. Five of the 17 observational studies evaluating the performance of PSP as a biomarker of infection in adult patients were finally included in the analysis (Fig. 1; Table 1). The main characteristics of these studies and levels of PSP, CRP and PCT among infected and non-infected patients are summarized in Tables 1 and 2 as well as in Additional file 3: Figure S1. Pooled individual data from 631 patients (371 infected and 260 non-infected) are presented in Additional file 1: Table S10. The biomarkers were measured in 479 patients admitted to the intensive care unit and in 152 admitted to the emergency room. The distributions of the three biomarkers significantly differed between infected and non-infected patients (Table 2; Additional file 1: Table S10, Additional file 3: Figure S3A and S3B). However, the overlap zone between the values of CRP and PCT among infected and non-infected patients was larger than that of PSP (Additional file 3: Figures S3A and S3B).

**Table 1:** Characteristics of included observational studies

Study	Data collection period	Country	*n* infection/non-infection	Clinical condition	Control group (non-infection)	PSP cut-off for infection/non-infection	AUC ROC if cut-off	Conclusion
Keel et al. [14]	January 2002 to September 2006	CH	49/14	ICU patients at day 5 post-admission for trauma	ICU patients without infection at day 5 post-admission for trauma	None	–	PSP levels in infected patients were > 15-fold increased over baseline
Llewelyn et al. [15]	August 2010 to January 2011	UK	88^a^/94	Unselected ICU or IMC patients	Unselected ICU or IMC patients without infection	30	0.93	PSP performed well as infection biomarker in patients with suspected infection at the time of admission
Gukasjan et al. [17]	August 2007 to February 2010	CH	88/43^b^	ICU patients with or without secondary peritonitis^b^	ICU patients after elective major abdominal surgery without secondary peritonitis^b^	None	–	PSP accurately predicted severity and outcome of severe infection
Klein et al. [18]	May 2012 to December 2012	CH	17/86	ICU patients two days post-cardiac surgery	ICU patients two days post-cardiac surgery without infection	48.1	0.77	PSP levels significantly associated with the presence of infection
Guadiana-Romualdo et al. [13]	October 2013 to November 2013	E	129/23	Unselected ER patients	Unselected ER patients without infection	41.5	0 84	PSP performed well as infection biomarker

CH, Switzerland; UK, United Kingdom; E, Spain; ICU, intensive care unit; IMC, Intermediate Care; ER, Emergency Room

^a^One subject from the infection group had an undetermined infection status in the original data set and was excluded from the biomarker analysis

^b^We received raw data from additional 43 control patients admitted after abdominal surgery but without suspicion/evidence of peritonitis whose characteristics were not included in the original publication (see Additional file 5: Supplemental material for details on the patients characteristics and biomarker levels of this control group)

**Table 2:** Characteristics of the patients’ population for each study

Publication	Age^a^	Males (%)	No infection PSP^a^ ng/ml	Infection PSP^a^ ng/ml	No infection CRP^a^ mg/l	Infection CRP^a^ mg/l	No infection PCT^a^ ng/ml	Infection PCT^a^ ng/ml
Keel et al. [14]	35 [21.5, 50.5]	48 (76%)	19.58 [12.79, 28.96]	52 [18.98, 170.5]	102 [70.5, 128.75]	113 [62, 162]	0.38 [0.25, 0.54]	0.54 0.34, 1.2]
Llewelyn [15]	66 [53, 75]	108 (60%)	17.8 [11.7, 29.7]	107.75 [49.08, 247]	10.6 [3.4, 26.5]	144.65 [104.97, 203.05]	0.2 [0.1, 0.8]	3.1 [0.8, 8.83]
Gukasjan et al. [17]	61 [50, 71]	75 (55%)	15.2 [11.21, 23.24]	125.25 [25.73, 414.38]	51.3 [32.7, 86.4]	222.8 [144.25, 286.55]	0.1 [0.03, 0.2]	1.07 [0.28, 6.05]
Klein et al. [18]	67 [54, 75]	#	22.6 [13.77, 46.7]	51.68 [33.18, 76.4]	125 [92.25, 157.75]	132.5 [106.25, 188.75]	#	#
Guadiana-Romualdo et al. [13]	65.5 [46.75, 78.25]	89 (59%)	22.7 [17.2, 30.35]	73 [33.2, 203]	48 [19, 135]	130 [67, 210]	0.08 [0.06, 0.15]	0.54 [0.23, 2.45]

^a^Age and biomarker levels are expressed as medians [interquartile range]. # Klein et al. reported neither gender nor PCT values

While a PSP *I*^2^ of 42% indicates only moderate heterogeneity in PSP ability to detect infection, the intercept *I*^2^ of 96% indicates an important heterogeneity in baseline risk of infection among the studies (Additional file 3: Figure S4 and Additional file 1: Table S5). Despite the slight asymmetry with extreme value given by the PSP effect in the study of Guadiano-Romulado [6], the classical Egger test did not reject the null symmetry hypothesis corresponding to ‘no bias in publications’ (Additional file 3: Figure S5).

### Diagnosing infection in hospitalized adult patients using single biomarkers and the ‘single-fit’ fixed-effect model

**Fig. 2:**
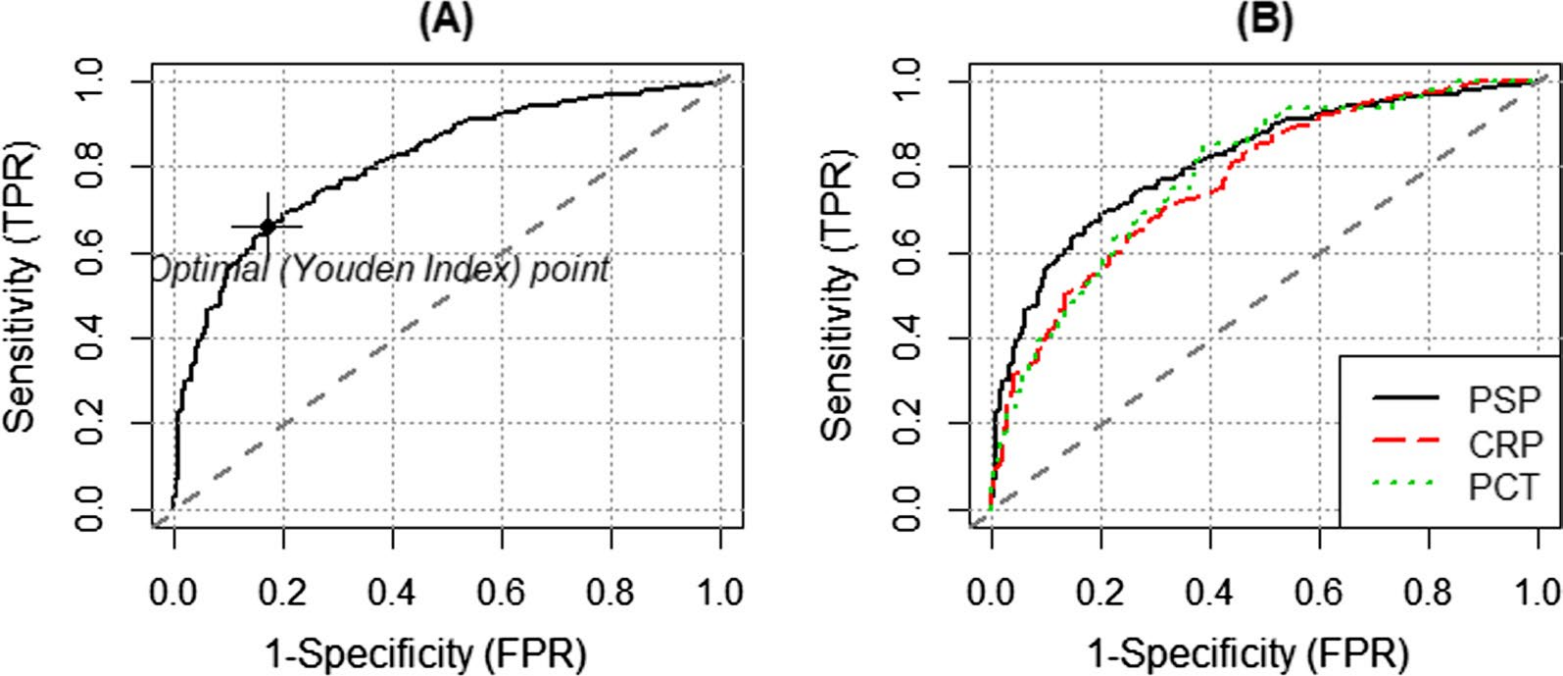
ROC curve analysis of PSP (**A**) compared to the ones of CRP (red) and PCT (green) (**B**) for the detection of infection in adult hospitalized patients. The cut-off values of the biomarkers were determined by the Youden’s index as (**A**) PSP 44.18 ng/ml with the corresponding ROC AUC = 0.81 and AUC 95% CI (0.78, 0.85); (**B**) CRP 99.05 mg/l with AUC 0.77 (0.73, 0.80) and PCT 0.20 ng/ml with AUC 0.78 (0.74, 0.82)

PSP performed slightly better than CRP and PCT for detecting infection among adult hospitalized patients (*p* = 0.044 and *p* = 0.010, DeLong test, Table 3). In a first step, using the Youden’s index [25] giving equal weight to both sensitivity and specificity, we identified an optimal PSP cut-off value of 44.18 ng/ml that best diagnoses infection with an ROC AUC of 0.81 (95% CI 0.78–0.85) (Fig. 2A; Table 3). Using that threshold, we calculated the corresponding positive [0.85 (0.81–0.89)] and negative [0.63 (0.58–0.68)] predictive values for the detection of infection. The same methods were applied to CRP and PCT. Their respective ROC AUC values computed threshold values of 99.05 mg/l and 0.2 ng/ml for CRP and PCT, respectively, were lower: CRP 0.77 [0.73–0.80] and PCT 0.78 [0.74–0.82] (Fig. 2B; Table 3). Additional file 3: Figure S6 and Additional file 1: Table S9 display the PSP, CRP and PCT AUC ROC values using the defined cut-offs presented in Table 3 and applied to each individual datasets of the five eligible studies.

**Table 3:** Performance of each biomarker in predicting infection

Biomarker (*n* =)	With/without infection	Youden’s index on predicted probabilities	Biomarker’ cut-off values for Youden’s index	AUC [95%CI) (SE)	Sensitivity (95%CI)	Specificity (95%CI)	PPV (95%CI)	NPV (95%CI)	PLR (95%CI)	PLR (95%CI)
PSP [ng/ml] *n* = 631	369/262	0.49	44.18	0.81 [0.78, 0.85] (0.017)	0.66 [0.61, 0.71]	0.83 [0.78, 0.88]	0.85 [0.81, 0.89]	0.63 [0.58, 0.68]	3.95 [2.99, 5.23]	0.40 [0.35, 0.47]
CRP [mg/ml] *n* = 629	369/260	0.39	99.05	0.77 [0.73, 0.80] (0.019)	0.70 [0.66, 0.75]	0.69 [0.63, 0.74]	0.76 [0.72, 0.81]	0.62 [0.56, 0.67]	2.35 [1.92, 2.87]	0.44 [0.38, 0.53]
PCT [ng/ml] *n* = 527	175/352	0.44	0.20	0.78 [0.74, 0.82] (0.022)	0.83 [0.79, 0.87]	0.62 [0.54, 0.69]	0.81 [0.78, 0.85]	0.64 [0.56, 0.71]	2.14 [1.77, 2.60]	0.27 [0.21, 0.36]

Using paired DeLong test on 527 patients for whom all three biomarkers are available, the *p* values for the null hypothesis ‘true difference in AUC is 0’ are 0.010 and 0.044 for comparisons PSP versus PCT and PSP versus CRP respectively (without adjustment for multiple testing)

PPV and NPV stand for positive, respectively, negative predicting value, when PLR and NLR for positive, respectively, negative likelihood ratio

### Diagnosing infection in hospitalized adult patients using combinations of biomarkers

In a second step, we determined whether various combinations of biomarkers would improve the performance of infection detection among adult hospitalized patients (Additional file 3: Figure S7 and Additional file 1: Table S11). The model combining PSP with CRP was better than the one combining PSP with PCT. When combining PSP to CRP, the performance increased to an ROC AUC of 0.90 (0.87, 0.92), with a sensitivity of 0.81 (0.77, 0.85), specificity 0.84 (0.79, 0.90), PPV 0.91 (0.88, 0.94) and NPV 0.69 (0.63, 0.75). Adding PCT to the PSP + CRP model did not further improve performance.

## Discussion

Since its first description as a potential infection biomarker in patients after trauma [14], pancreatic stone protein has repeatedly been shown to perform better than CRP and at least as well as PCT in identifying patients with infection [13–15, 17, 18]. However, in contrast to PCT [6, 26], no cut-off threshold value has yet been defined for its eventual clinical use.

We were able to obtain raw data from five small observational studies and did a meta-analysis at the individual patient level to explore the performance of PSP in detecting infection. The eligible studies were performed in different countries across Europe and included acutely ill patients from ERs or ICUs. The resulting cohort of 631 hospitalized adult patients encompassed an important proportion (42%) of patients without infection, which makes it the largest analysis of this nature on PSP.

Because we observed low heterogeneity of PSP effect despite strong heterogeneity in the baseline risk of infection among studies, we decided not to adjust for heterogeneity in the models presented in the main manuscript, because it may not be of clinical relevance (see Additional file 5: Supplemental section for models adjusting for heterogeneity). In the trade-off—adjusting for heterogeneity and improving performance of the biomarker versus addressing a clinical need and computing a unique biomarker threshold—we chose to omit adjustment for heterogeneity, especially as we wanted to determine a PSP cut-off value and compare it to those of PCT and CRP currently largely used at the bedside [6–10].

We applied a stepwise approach to evaluate the performance of PSP and compared it to those of CRP and PCT for detecting infection in adult hospitalized patients presenting to the ER or the ICU. In all explored scenarios, PSP achieved a statistically significant better performance compared to CRP and PCT. Using the Youden’s index approach, we found a PSP cut-off value of 44.12 ng/ml, which is approximately four-fold higher than the upper value previously determined in 61 healthy adult volunteers (min. 4.0, max 18.3, median 10.8 ng/ml) [27]. Using the same methodology, we found a CRP cut-off value of 99.5 mg/l and a PCT cut-off value of 0.2 ng/ml. Moreover, because there is much less overlap in PSP levels between infected and non-infected patients, PSP could guide decisions to start antibiotic treatment, as opposed to PCT that is currently rather used in ICU patients for antibiotic de-escalation [8, 11, 28].

The inclusion of post-trauma and post-operative populations with a very low baseline risk of infection might have limited the ability of CRP and PCT to diagnose infection. Previous studies and meta-analysis exploring the diagnostic value of PCT for the detection of patients with infection have indeed reported that PCT accuracy and cut-off values are highly dependent on the clinical settings and the baseline risks for infection [6, 26]. On the other hand, this might simply have highlighted the possible advantage of PSP over PCT and CRP in particular situations characterized by severe non-infectious systemic inflammatory states. Two studies including patients post-burn trauma recently confirmed this observation [29, 30]. Interestingly, PSP levels rose up to 72 h before the clinical diagnosis of infection, confirming its potential role as an early biomarker of infection.

We further explored the value of biomarkers combinations. Combining PSP with CRP increased the accuracy of detecting infection from an AUC 0.81 to 0.90, a value that is usually considered as very good [31] and among the highest AUCs reported for this setting [4, 8, 9]. Interestingly, adding CRP to PSP markedly increases the sensitivity while not decreasing the specificity achieved by PSP alone (Additional file 1: Table S11).

This study has several strengths, which makes its results potentially generalizable. First, it was possible to include individual patient data of all published studies that evaluated the value of PSP to detect infection in hospitalized patients before March 2019. Second, it encompasses important clinical settings—ICUs and ERs—where early detection of infection is of the utmost importance in order to guide rapid management [1]. Third, the raw data come from studies performed in several centres in the UK, Spain and Switzerland, reducing the risk of centre-effect bias. On the other hand, the measurement of PSP level was consistent throughout all studies, minimizing the risk of methodological bias. Finally, yet importantly, a balanced proportion of infected and non-infected patients was included in this analysis.

The main limitation of our study is the heterogeneity of the included populations, mixing community- and healthcare-acquired infections: some populations were included from medical emergency admissions with the goal to confirm infection at admission, while other patients were trauma or surgical patients who were likely to develop infection during hospitalization. This translates into an important heterogeneity in baseline risk of infection among the studies. Moreover, the five studies included patients for whom suspected or documented infection was the main reason of referral, which may limit the generalization of the findings in other circumstances. In addition, because only one study considered patients in the ER, we could not investigate subgroup of patients from ERs versus ICUs.

A further important limitation of primary research in infection biomarker evaluation is the lack of an objective gold standard definition of infection. Nevertheless, each of the five eligible studies set out clear criteria and procedures, all consistent with one another, using a synthesis of available clinical, radiological and microbiological evidences to assign each case as infection or not. Furthermore, the fact that we find low heterogeneity of PSP effect across the range of studies performed is reassuring that our fundamental observation is not undermined by variability in case definition. Moreover, the ability of PSP to diagnose infection is confirmed across all studies.

Our study opens new possibilities for the future use of PSP, including being used as a tool to optimize antibiotic stewardship program, as a biomarker to guide the decision to start antibiotics, or as inclusion or stratification criteria for future studies of severe infections. PSP is now available at the point of care (POC) with a turnaround time of less than 5 min [12] making it highly suitable for situations where time to antibiotic is essential.

The next step would be to validate these results in independent cohorts of patients. As baseline risk of infection impacts the performance of a diagnostic biomarker and its corresponding threshold, the validation process would require to perform independent studies in selected settings (primary care, ER, ICU), each including at best patients with a homogenous baseline risk of infection. Such a thorough validation process has been performed for PCT [6, 26] in large independent cohorts of patients, each with different baseline risks of infection and is still required before PSP to be routinely used in the clinic.

The benefit of serial measurement of PSP for the early detection of sepsis in intensive care unit patients has been evaluated in a prospective trial using the new POC technology [32]. This patient population would be the first ideal cohort to validate the defined threshold in a homogenous patient population as well as the value of combining PSP to CRP. Both biomarkers can be now measured using point-of-care technologies; however, correction factors will be required while using new measurement assays and technologies. Finally, our methodology can be used to further evaluate the value of PSP in detecting the severity of the infection as well as its prognosis and to define for thresholds for that purpose as well.

## Conclusions

This study confirms that PSP is a promising biomarker to detect infection in hospitalized patients. Using a cut-off value of 44.18 ng/ml, PSP performs better than CRP or PCT across the considered studies. The combination of PSP with CRP further enhance its accuracy. However, further and especially prospective studies are needed to confirm its utility and safety in the daily clinical use, especially as a potential biomarker to guide the initiation of antibiotics.

## Supplementary Information

**Additional file 1.** Supplemental Tables.

**Additional file 2.** Approved Study Protocol.

**Additional file 3.** Supplemental Figures.

**Additional file 4.** Supplemental Methods.

**Additional file 5.** Supplemental Material.

## Data Availability

The corresponding author has access to all data included into the analysis. Requests should be submitted to the corresponding author in the first instance.

## Abbreviations

PSP: Pancreatic stone protein
PCT: Procalcitonin
CRP: C-reactive protein
ICU: Intensive care unit
ER: Emergency room
POC: Point of care
