# Supplementary material for: Accuracy of pancreatic stone protein for the diagnosis of infection in hospitalized adults: a systematic review and individual patient level meta-analysis
Source: Crit Care. 2021 May 28;25:182. doi: 10.1186/s13054-021-03609-2 (PMC8164316; doi:10.1186/s13054-021-03609-2)
Supplement: Supplementary file 1 — Additional file 1. Supplemental Tables. [file 13054_2021_3609_MOESM1_ESM.docx]

**Supplemental Table 1: PRISMA Checklist**

| **Section/topic** | **#** | **Checklist item** | **Reported on page #** |
| --- | --- | --- | --- |
| **TITLE** | | |  |
| Title | 1 | Identify the **report as a systematic review, meta-analysis, or both**. | 1 |
| **ABSTRACT** | | |  |
| Structured summary | 2 | Provide a structured summary including, as applicable: background; objectives; data sources; study eligibility criteria, participants, and interventions; study appraisal and synthesis methods; results; limitations; conclusions and implications of key findings; systematic review registration number. | 2 |
| **INTRODUCTION** | | |  |
| Rationale | 3 | Describe the rationale for the review in the context of what is already known. | 3-4 |
| Objectives | 4 | Provide an explicit statement of questions being addressed with reference to participants, interventions, comparisons, outcomes, and study design (PICOS). | 3-4 |
| **METHODS** | | |  |
| Protocol and registration | 5 | Indicate if a review protocol exists, if and where it can be accessed (e.g., Web address), and, if available, provide registration information including registration number. | Yes; provided as appendix |
| Eligibility criteria | 6 | Specify study characteristics (e.g., PICOS, length of follow-up) and report characteristics (e.g., years considered, language, publication status) used as criteria for eligibility, giving rationale. | 6 |
| Information sources | 7 | Describe all information sources (e.g., databases with dates of coverage, contact with study authors to identify additional studies) in the search and date last searched. | 6 |
| Search | 8 | Present full electronic search strategy for at least one database, including any limits used, such that it could be repeated. | 5 |
| Study selection | 9 | State the process for selecting studies (i.e., screening, eligibility, included in systematic review, and, if applicable, included in the meta-analysis). | 5; 8 |
| Data collection process | 10 | Describe method of data extraction from reports (e.g., piloted forms, independently, in duplicate) and any processes for obtaining and confirming data from investigators. | 5-6; 8 |
| Data items | 11 | List and define all variables for which data were sought (e.g., PICOS, funding sources) and any assumptions and simplifications made. | 6-7 |

| Risk of bias in individual studies | 12 | Describe methods used for assessing risk of bias of individual studies (including specification of whether this was done at the study or outcome level), and how this information is to be used in any data synthesis. | 5-6 |
| --- | --- | --- | --- |
| Summary measures | 13 | State the principal summary measures (e.g., risk ratio, difference in means). | 8-9 |
| Synthesis of results | 14 | Describe the methods of handling data and combining results of studies, if done, including measures of consistency (e.g., *I^2^*) for each meta-analysis. | 6-7 |
| Risk of bias across studies | 15 | Specify any assessment of risk of bias that may affect the cumulative evidence  (e.g., publication bias, selective reporting within studies). | 5-6, 11-12;  Suppl. Fig. 5, Suppl. Table 2 |
| Additional analyses | 16 | Describe methods of additional analyses (e.g., sensitivity or subgroup analyses, meta-regression), if done, indicating which were pre-specified. | 7 |
| **RESULTS** | | |  |
| Study selection | 17 | Give numbers of studies screened, assessed for eligibility, and included in the review, with reasons for exclusions at each stage, ideally with a flow diagram. | 8; Fig 1 |
| Study characteristics | 18 | For each study, present characteristics for which data were extracted (e.g., study size, PICOS, follow-up period) and provide the citations. | 8; Tab-1 |
| Risk of bias within studies | 19 | Present data on risk of bias of each study and, if available, any outcome level assessment (see item 12). | Limitation paragraph pages 11-12  Suppl. Table 2 |
| Results of individual studies | 20 | For all outcomes considered (benefits or harms), present, for each study: (a) simple summary data for each intervention group (b) effect estimates and confidence intervals, ideally with a forest plot. | Figure 2  Table 2 |
| Synthesis of results | 21 | Present results of each meta-analysis done, including confidence intervals and measures of consistency. | Supplemental Table 4, 5, 6, 7,8 and 9 |
| Risk of bias across studies | 22 | Present results of any assessment of risk of bias across studies (see Item 15). | Limitation paragraph pages 11-12 |
| Additional analysis | 23 | Give results of additional analyses, if done (e.g., sensitivity or subgroup analyses, meta-regression [see Item 16]). | 9 |

| **DISCUSSION** | | |  |
| --- | --- | --- | --- |
| Summary of evidence | 24 | Summarize the main findings including the strength of evidence for each main outcome; consider their relevance to key groups (e.g., healthcare providers, users, and policy makers). | 10 |
| Limitations | 25 | Discuss limitations at study and outcome level (e.g., risk of bias), and at review-level (e.g., incomplete retrieval of identified research, reporting bias). | 11-12 |
| Conclusions | 26 | Provide a general interpretation of the results in the context of other evidence, and implications for future research. | 12 |
| **FUNDING** | | |  |
| Funding | 27 | Describe sources of funding for the systematic review and other support (e.g., supply of data); Role of funders for the systematic review. | 12 |

**Supplemental Table 2: The Cochrane Risk of Bias Diagram.**

| Random sequence generation (selection bias) | N/A | Prospective observational study |
| --- | --- | --- |
| Allocation concealment (selection bias) | N/A | Prospective observational study |
| Blinding of participants and personnel (performance bias) | N/A | Prospective observational study |
| Blinding of outcome assessment (patient-reported outcomes) (detection bias) | Low risk | For every included study, the patient’s infection status was unknown at inclusion / admission to ICU / ED.  Definition of infection was clearly stated in each of the eligible publications.  In two studies, categorization of subjects (infection versus non-infection) was made independently by two investigators blind to the biomarkers results |
| Incomplete outcome data addressed (attrition bias) | Low risk | PSP data were available for all patients included and reported in the eligible studies |
| Selective reporting (reporting bias) | Low / Medium risk | We use the totality of the raw data from all eligible studies that were identified during the screening  However, most of the eligible observational studies recruited patients for whom an infection was either suspected or documented |

**Supplemental Table 3: Definition of infection for each of the five eligible studies.**

| **Study** | **Clinical Condition** | **Infection definition** |
| --- | --- | --- |
| Keel et al. (2009) | ICU patients at day 5 post trauma | Patients were categorized *post hoc* according to their clinical data: a) without infection, b) with local infection, and c) with sepsis. Patients with local infection and sepsis were pooled together and compared to patients without infection.  Presence of any kind of infection defined as a positive bacterial tissue culture or a positive blood culture. |
| Llewelyn et al. (2013) | Unselected ICU or IMC patients | Presence of infection defined as SIRS plus either positive microbiology or radiology or clinical presentation  Non-infected SIRS defined as SIRS associated with an established underlying non-infective diagnosis and no reason to suspect any ongoing infection.  Categorization of subjects was made independently by two investigators blind to the biomarkers results  Patients classified as indeterminate were excluded from meta-analysis |
| Gukasjan et al. (2013) | ICU patients with or without secondary peritonitis | Presence of infection defined as proven diagnosis of secondary peritonitis according to the Mannheim Peritonitis Index |
| Klein et al. (2015) | ICU patients two days post cardiac surgery | Presence of postoperative infection at days 2 post cardiac surgery defined according to definition of infection and sepsis by Levy, 2003 (Levy et al, 2001 SCCM/ESICM/ACCP/ATS/SIS International Sepsis Definitions Conference. *Critical care medicine* 2003; 31: 1250-1256). |
| Guadiana-Romualdo et al. (2017) | Unselected ER patients | Patients with clinically relevant positive bacterial microbiological cultures collected within 48 h of enrolment and patients with strong evidence for infection in the absence of positive cultures (radiographic evidence [computed tomography scan, chest X-ray, etc.] or physical examination findings strongly suggesting bacterial infection in the absence of positive cultures)  Patient classification was determined by using a majority rule among two physicians, all blinded to biomarker results |

ICU: Intensive Care Unit; IMC: Intermediate Care; ER: Emergency Room; SIRS: systemic inflammatory response syndrome

**Supplemental Table 4:** **Comparison of the PSP meta-analysis models for predicting infection status within the five eligible studies.**

The reported p-value of fully stratified fit against the considered model stands for the hypothesis, ‘the variance of the concerned random effect is zero’. When testing the *‘fully-stratified*’ random effect model against the *mixed-effects model with random intercept*, the p-value is 0.8. However, when testing mixed-effects model versus fixed-effects model “the variance of intercept and PSP random effect are equal to zero” is rejected with p-value < 0.0001. According to Steyerberg et al (2019) the log-likelihood ratio test is distributed as 50:50 mixture of chi-squared distributions with 6 and 10 degrees of freedom in first case and 15 and 10 in second case.

| **Model variant** | **Baseline risk** | **Predictor effect** | **Estimates** | **-2 log-likelihood** | **p-value of fully stratified fit against the considered model** |
| --- | --- | --- | --- | --- | --- |
| ‘*Fully stratified*’  Random-effect | Per study | Per study | See Supplemental Tables 5A and 6 | 592.0 |  |
| Mixed-effect | Per study | Common | See Supplemental Table 5B and Supplemental Table 7 | 593.9 | 0.8 |
| Fixed effect | Common | Common | See main manuscript Table 4 (PSP raw), Supplemental Table 5B and Supplemental Table 9 | 694.5 | p < 0.0001 |

**Supplemental Table 5:** **The multivariable logistic regression models for predicting infection status using PSP.**

(A) ‘*Fully-stratified’* random-effect model. (B) Mixed-effects model with random intercept. (C) Fixed effects model. Results of the fixed-effects model are presented in the manuscript.

**Supplemental Table 5A: Estimates computed for the *‘fully-stratified’* random-effect model**.

We performed a two-stage meta-analysis by first fitting logistic regression model in every study separately and by then fitting the meta-analysis to the estimates obtained in the first stage. The between- versus within-study heterogeneity is summarized with *I^2^* estimates.

| **Study** | **Intercept (SE)** | **PSP (SE)** |
| --- | --- | --- |
| Keel et al. (2009) | 1.84 (0.59) | 2.15 (1.25) |
| Llewelyn et al (2013) | 0.65 (0.29) | 3.38 (0.65) |
| Gukasjan et al. (2013) | 0.94 (0.28) | 1.70 (0.54) |
| Klein et al. (2015) | -1.02 (0.39) | 1.73 (0.88) |
| Guadiana-Romualdo et al. (2017) | 5.65 (1.49) | 9.99 (3.13) |
| Pooled estimates | 1.02 (0.65) | 2.58 (0.59) |
| Estimated *τ^2^* | 3.68 | 0.54 |
| *I^2^* | 96% | 42% |
| *H^2^* | 23.31 | 1.72 |
| 95% Prediction interval | [-1.95, 4] | [0.44, 4.71] |

**Supplemental Table 5B: Estimates computed for the mixed-effect model.**

|  | **Estimate [95% CI]** | **P-value** |
| --- | --- | --- |
| Intercept | 1.12 [-0.12, 2.38] | 0.0337 |
| PSP | 2.75 [2.08, 3.53] | < 0.0001 |
| n= | 631 |  |
| Standard deviation of random intercept | 1.12 |  |

**Supplemental Table 5C**: **Estimates computed for the fixed-effect model.**

|  | **Estimate [95% CI]** | **P-value** |
| --- | --- | --- |
| Intercept | 1.03 [0.74, 1.35] | < 0.0001 |
| PSP | 2.74 [2.10, 3.46] | < 0.0001 |
| n= | 631 |  |
|  |  |  |

**Supplemental Table 6: AUC of the ROC curves for detecting infection using the respective PSP cutoffs determined for each study by the *‘fully stratified’* random-effect model.**

| **Study** | **With/ without Infection** | **Cutoff ng/ml** | **AUC [95% CI] [SE]** | **SEN**  **[95% CI]** | **SPE**  **[95% CI]** | **PPV**  **[95% CI]** | **NPV**  **[95% CI]** | **PLR**  **[95% CI]** | **NLR**  **[95% CI]** |
| --- | --- | --- | --- | --- | --- | --- | --- | --- | --- |
| All | 371/260 | Determined for each study | 0.88  [0.85, 0.90] [0.014] | 0.78 [0.73, 0.82] | 0.82 [0.78, 0.87] | 0.86 [0.82, 0.90] | 0.72 [0.67, 0.77] | 4.42 [3.38, 5.77] | 0.27 [0.22, 0.33] |
| Keel et al. (2009) | 49/14 | 31.33 | 0.72 [0.58, 0.87]  [0.074] | 0.59 [0.45, 0.73] | 0.79 [0.57, 1.00] | 0.91 [0.80, 1.01] | 0.35 [0.19, 0.52] | 2.76 [0.99, 7.73] | 0.52 [0.34, 0.80] |
| Llewelyn et al. (2013) | 86/96 | 35.15 | 0.89 [0.84, 0.94]  [0.025] | 0.88 [0.82, 0.95] | 0.82 [0.75, 0.90] | 0.82 [0.74, 0.90] | 0.89 [0.82, 0.95] | 4.99 [3.22, 7.73] | 0.14 [0.08, 0.26] |
| Gukasjan et al. (2013) | 88/43 | 30.77 | 0.84 [0.77, 0.91]  [0.037] | 0.67 [0.57, 0.77] | 0.93 [0.85, 1.01] | 0.95 [0.90, 1.00] | 0.58 [0.46, 0.70] | 9.61 [3.19, 28.90] | 0.35 [0.26, 0.48] |
| Klein et al (2015) | 17/86 | 25.96 | 0.70 [0.58, 0.83]  [0.065] | 0.82 [0.64, 1.00] | 0.55 [0.44, 0.65] | 0.26 [0.14, 0.38] | 0.94 [0.87, 1.01] | 1.82 [1.32, 2.50] | 0.32 [0.11, 0.92] |
| Guadiana-Romualdo et al.  (2017) | 129/23 | 42.50 | 0.84 [0.77, 0.90]  [0.033] | 0.67 [0.59, 0.76] | 0.96 [0.87, 1.04] | 0.99 [0.97, 1.01] | 0.34 [0.23, 0.46] | 15.51 [2.27, 105.87] | 0.34 [0.26, 0.44] |

PPV and NPV stand for positive, respectively negative predicting value, when PLR and NLR for positive, respectively negative likelihood ratio.

**Supplemental Table 7: AUC of the ROC curves for detecting infection using the respective PSP cutoffs determined for each study using the mixed-effect model with random intercept.**

| **Study** | **With/ without Infection** | **PSP**  **Cutoff**  **ng/ml** | **AUC [95% CI] [SE]** | **Sensitivity [95% CI]** | **Specificity [95% CI]** | **PPV [95% CI]** | **NPV [95% CI]** | **PLR [95% CI]** | **NLR [95% CI]** |
| --- | --- | --- | --- | --- | --- | --- | --- | --- | --- |
| All | 371/260 | Determined for each study | 0.87 [0.84, 0.90]  [0.014] | 0.82 [0.78, 0.86] | 0.78 [0.72, 0.82] | 0.84  [0.80, 0.88] | 0.76 [0.70, 0.81] | 3.65 [2.90, 4.59] | 0.23 [0.18, 0.29] |
| Keel et al. (2009) | 49/14 | 29.36 | 0.72 [0.58, 0.87]  [0.074] | 0.59 [0.45, 0.73] | 0.79 [0.57, 1.00] | 0.91 [0.80, 1.01] | 0.35 [0.19, 0.52] | 2.76 [0.99, 7.73] | 0.52 [0.34, 0.80] |
| Llewelyn et al. (2013) | 86/96 | 35.00 | 0.89 [0.84, 0.94]  [0.025] | 0.88 [0.82, 0.95] | 0.82 [0.75, 0.90] | 0.82 [0.74, 0.90] | 0.89 [0.82, 0.95] | 4.99 [3.22, 7.73] | 0.14 [0.08, 0.26] |
| Gukasjan et al. (2013) | 88/43 | 31.45 | 0.84 [0.77, 0.91]  [0.037] | 0.67 [0.57, 0.77] | 0.93 [0.85, 1.01] | 0.95 [0.90, 1.00] | 0.58 [0.46, 0.70] | 9.61 [3.19, 28.90] | 0.35 [0.26, 0.48] |
| Klein et al. (2015) | 17/86 | 25.84 | 0.70 [0.58, 0.83]  [0.065] | 0.82 [0.64, 1.00] | 0.55 [0.44, 0.65] | 0.26 [0.14, 0.38] | 0.94 [0.87, 1.01] | 1.82 [1.32, 2.50] | 0.32  [0.11, 0.92] |
| Guadiana- Romualdo et al. (2017) | 129/23 | 43.50 | 0.84 [0.77, 0.90]  [0.033] | 0.67 [0.59, 0.76] | 0.96 [0.87, 1.04] | 0.99 [0.97, 1.01] | 0.34 [0.23, 0.46] | 15.51 [2.27, 105.87] | 0.34 [0.26, 0.44] |

PPV and NPV stand for positive, respectively negative predicting value, when PLR and NLR for positive, respectively negative likelihood ratio.

**Supplemental Table 8**: **Continuous covariate odds ratios for the diagnostic of infection for each joint model combining biomarkers**.
Models were computed on the 527 patients with available PCT values, as Klein *et al.* did not report any PCT values.

| **Covariate** | **OR** | **OR 95% CI** | **p-value** |
| --- | --- | --- | --- |
| Joint model with PSP and CRP |  |  |  |
| PSP ng/ml | 1.010 | 1.006 – 1.014 | <0.001 |
| CRP mg/l | 1.019 | 1.015 – 1.023 | <0.001 |
| Joint model with PSP and PCT |  |  |  |
| PSP ng/ml | 1.014 | 1.010 – 1.019 | <0.001 |
| PCT ng/ml | 1.043 | 0.970 – 1.123 | 0.255 |
| Joint model with PSP, CRP and PCT |  |  |  |
| PSP ng/ml | 1.009 | 1.005 – 1.014 | <0.001 |
| CRP mg/l | 1.019 | 1.015 – 1.023 | <0.001 |
| PCT ng/ml | 1.015 | 0.964 – 1.070 | 0.571 |

**Supplemental Table 9: AUC of the ROC curves computed for each single dataset for detecting infection using the PSP cutoff at 44.18 ng/ml determined by the fixed-effect model.**

The corresponding ROC curves are shown in Supplemental Fig.6A

| **Study** | **With/ without Infection** | **AUC [95% CI] [SE]** | **Sensitivity [95% CI]** | **Specificity [95% CI]** | **PPV [95% CI]** | **NPV [95% CI]** | **PLR [95% CI]** | **NLR [95% CI]** |
| --- | --- | --- | --- | --- | --- | --- | --- | --- |
| All | 371/260 | 0.81 [0.78, 0.85] [0.017] | 0.66  [0.62, 0.71] | 0.83 [0.79, 0.88] | 0.85 [0.81, 0.89] | 0.64 [0.59, 0.69] | 3.95 [2.99, 5.23] | 0.40 [0.35, 0.47] |
| Keel et al. (2009) | 49/14 | 0.67  [0.49, 0.84] [0.09] | 0.55 [0.41, 0.69] | 0.79 [0.57, 1.00] | 0.90 [0.79, 1.01] | 0.33 [0.17, 0.49] | 2.57 [0.91, 7.24] | 0.57 [0.38, 0.86] |
| Llewelyn et al. (2013) | 86/96 | 0.83 [0.76, 0.91] [0.039] | 0.81  [0.73, 0.90] | 0.85 [0.78, 0.92] | 0.83 [0.75, 0.91] | 0.84 [0.76, 0.91] | 5.58 [3.40, 9.15] | 0.22  [0.14, 0.34] |
| Gukasjan et al. (2013) | 88/43 | 0.77 [0.68, 0.86] [0.045] | 0.61 [0.51, 0.71] | 0.93 [0.85, 1.01] | 0.95 [0.89, 1.00] | 0.54 [0.43, 0.65] | 8.79 [2.92, 26.53] | 0.41 [0.32, 0.55] |
| Klein et al. (2015) | 17/86 | 0.63 [0.47, 0.80] [0.084] | 0.53  [0.29, 0.77] | 0.73 [0.64, 0.83] | 0.28 [0.12, 0.44] | 0.89 [0.81, 0.96] | 1.98 [1.12, 3.50] | 0.64 [0.38, 1.08] |
| Guadiana-Romualdo et al. (2017) | 129/23 | 0.81  [0.72, 0.89] [0.042] | 0.66 [0.58, 0.74] | 0.96  [0.87, 1.04] | 0.99 [0.97, 1.01] | 0.33 [0.22, 0.45] | 15.15 [2.22, 103.47] | 0.36  [0.28, 0.46] |

PPV and NPV stand for positive, respectively negative predicting value, when PLR and NLR for positive, respectively negative likelihood ratio.

**Supplemental Table 10: Pooled individual data from the five studies included in the analysis**

|  | **All patients#**  **n=631** | **Non-infected Patients**  **n=260** | **Infected patients**  **n=371** |
| --- | --- | --- | --- |
| **DEMOGRAPHICS** [available n=] |  |  |  |
| Age [n=628] | 63 [49, 74] | 63 [51, 73.25] | 63 [47, 74] |
| Male [n=528]* | 319 [60%] | 102 [59%] | 217 [62%] |
| Outcome death [n=393] | 50 [13%] | 7 [4%] | 43 [19%] |
| **CLINICAL SETTING** |  |  |  |
| ICU | 479 | 238 [49.7%] | 241 [50.3%] |
| Emergency | 152 | 23 [15.1%] | 129 [84.9%] |
| **BIOMARKERS** [available n=] |  |  |  |
| PSP ng/ml [n=631] | 36.6 [17.7, 127] | 19.2 [12.6, 33.57] | 81.5 [30, 237.5] |
| PCT ng/ml [n=527]* | 0.5 [0.15, 2.41] | 0.15 [0.08, 0.5] | 0.9 [0.29, 4.4] |
| CRP mg/l [n=629] | 113 [49, 180] | 58.25 [15.85, 120] | 150 [82.7, 229.55] |

*Klein et al. reported neither gender nor PCT values

**Supplemental Table 11: Performance of models combining biomarkers for the prediction of infection using biomarkers as continuous covariates.**

| **Model specification**  **(n=)** | **Youden’s index on predicted probabilities** | **AUC**  **[95%CI]**  **(SE)** | **Sensitivity**  **[95%CI]** | **Specificity**  **[95%CI** | **PPV**  **[95%CI]** | **NPV**  **[95%CI]** | **PLR**  **[95%CI]** | **NLR**  **[95%CI]** |
| --- | --- | --- | --- | --- | --- | --- | --- | --- |
| -1.76 + 0.01*PSP + 0.02*CRP  (n=527) | 0.58 | 0.90 [0.87, 0.92] (0.014) | 0.82 [0.78, 0.86] | 0.85 [0.79, 0.90] | 0.91 [0.88, 0.94] | 0.70 [0.64, 0.76] | 5.30 [3.74, 7.53] | 0.21 [0.17, 0.27] |
| -0.35 + 0.01*PSP + 0.04*PCT  (n=527) | 0.55 | 0.83 [0.80, 0.87] (0.018) | 0.72 [0.68, 0.78] | 0.84 [0.78, 0.89] | 0.90 [0.86, 0.93] | 0.59 [0.54, 0.67] | 4.41 [3.14, 6.18] | 0.32 [0.27, 0.39] |
| -1.75 + 0.01*PSP + 0.02*CRP + 0.02*PCT  (n=527) | 0.58 | 0.90 [0.87, 0.92] (0.014) | 0.82 [0.78, 0.86] | 0.84 [0.79, 0.90] | 0.92 [0.88, 0.94] | 0.69 [0.63, 0.75] | 5.27 [3.71, 7.48] | 0.22 [0.18, 0.28] |

PPV and NPV stand for positive, respectively negative predicting value
