# Supplementary material for: Accuracy of pancreatic stone protein for the diagnosis of infection in hospitalized adults: a systematic review and individual patient level meta-analysis
Source: Crit Care. 2021 May 28;25:182. doi: 10.1186/s13054-021-03609-2 (PMC8164316; doi:10.1186/s13054-021-03609-2)
Supplement: Supplementary file 2 — Additional file 2. Approved Study Protocol. [file 13054_2021_3609_MOESM2_ESM.pdf]

Prüfplan/Protokoll HFV:
Weiterverwendung biologischen Materials und/oder gesundheitsbezogener Personendaten für die Forschung bei fehlender Einwilligung und Information nach Artikel 34 HFG

Prognostic Value of Pancreatic Stone Protein in Adult Patient: Systemic Review and Metaanalysis

(MetaPSP)

Josef Prazak MD, PhD[1], Yok-Ai Que MD, PhD, Prof[1]

(1) Department for Intensive Care, Bern University Hospital, University of Bern, Bern, Switzerland

Version 1.0; 18.06.2018

Name und Adresse der Investigators

Dr. med. Josef Prazak, PhD, Oberarzt
Department of Intensive Care, University Hospital Bern and University of Bern
Freiburgstrasse 10
3010 Bern
Switzerland
Tel.: +41 (0) 31 632 01 67
Email: josef.prazak@insel.ch

Principle investigator und Sponsor

Prof. Dr. med. Yok-Ai Que, PhD, Leitender Arzt
Department of Intensive Care, University Hospital Bern and University of Bern
Freiburgstrasse 10
3010 Bern
Switzerland
Tel.: +41 (0) 31 632 45 21
Email: yok-ai.que@insel.ch

Statistician

Dr. Armando Lenz, PhD
CTU Bern, University of Bern
Mittelstrasse 43
3012 Bern

Bestätigung der Projektleitung
Mit meiner Unterschrift bezeuge ich, dass sämtliche Angaben in diesem Prüfplan korrekt sind, und dass ich mich an meine gemachten Angaben und die nationale Gesetzgebung, namentlich den Datenschutz, halten werde.

Projektleiter:

Bern, 27.7.2018

Ort, Datum	Unterschrift

Abkürzungen

PSP	Pancreatic Stone Protein
ED	Emergency Department
ICU	Intensive Care Unit

1. Background

Sepsis and septic shock are critical conditions linked with high mortality and morbidity that required immediate action. Diagnosis of sepsis, assessment of its severity as well as its prognosis is however difficult.

Serum biomarkers may assist clinicians in risk stratification and decision-making processes. An ideal biomarker in patients with sepsis should improve early diagnosis and predict early deterioration toward organ failure and eventually death, thereby identifying patients requiring additional aggressive treatments. Owing to a lack of specificity or sensitivity or both [for example, C-reactive protein (CRP) and procalcitonin (PCT)] or to a narrow time window of expression [for example, interleukin-6 (IL-6) and IL-8], currently used biomarkers do not fulfill such requirements. Therefore, further efforts are needed to identify novel sepsis biomarkers

Pancreatic stone protein/regenerating protein (PSP/reg) is constitutively secreted by pancreatic acinar cells into pancreatic juice along with zymogens and is also secreted by subsets of intestinal and gastric cells. Although its precise physiological roles remain only partly defined, it appears to have protective functions by promoting cellular proliferative responses during beta-cell regenerative processes and epithelial repair. PSP/reg is upregulated during acute and chronic pancreatitis; in animals, its expression may be induced by stress conditions in the absence of any pancreatic inflammation.

PSP has been be used in several studies to diagnose sepsis characterize the severity of infection, and predict the outcome of patients with sepsis requiring ICU management, with ventilator-associated pneumonia or peritonitis.

2. Aim of the study

To perform systematic reviews and meta-analyses, first for the prognostic value of PSP for survival of patients, and second, for the diagnosis of sepsis.

3. Origin of Data

Data was collected by various research groups. We will receive individual patient data from eligible studies in a coded form, including baseline characteristics of patients, outcomes (sepsis or mortality), and laboratory values (PSP). We will receive the data from the first or last author of the eligible studies, i.e. Yok-Ai Que (1), Martin Llewelyn (2), Raphael Gukasjan (3), Marius Keel (4), Lukas Boeck (5), Luis García de Guadiana-Romulado (6), and Holger J. Klein (7). We will have no access to the code list of the individual studies, except for study (1), where the PI was first author.

4. Inclusion criteria

General inclusion criteria:

- Studies published both as abstracts and full-text articles.
- Studies using all study-designs, conducted in any study settings and regardless of sample size, study location, language of publication, and country of origin of test.
- Study in adults (≥18 years) performed on ICU/ED

For survival:

-

- Studies which employed PSP to predict survival in sepsis
-

For diagnosis of sepsis:
- Studies which employed PSP to diagnose sepsis
- Studies which employed PSP to assess severity of sepsis

5. Exclusion criteria

- reports from the manufacturer and package inserts which are subjected to overt conflict of interest
- Duplicate reports.
- Studies performed without informed consent of included participant

6. Begründung für den Antrag auf eine stellvertretende Einwilligung durch die zuständige Ethikkommission

We intend to meta-analyse data sets from about 500 patients included in 7 studies, which were conducted in years 2009-2015. Five of the studies include information on survival (1-5), and four of the studies include information about the prognosis and severity of sepsis (2,4,6,7). Four of the studies included patients from Switzerland (1,4,5,7). All patients have given an informed consent to participate on these studies; however the actual consent for further use of the patient data is missing. According to the demographic structure of involved patients, the probability, that up to one third of them died, is high. Therefore, it is very difficult and connected with disproportionately high effort to find the surviving patients or the relatives of patients who died.

In our intended study, there is absence of study-specific measures concerning the patients (retrospective design of already collected data from studies performed with consent). Moreover, our study can show important relationship between a relatively new infection marker and patient outcome and reveal its early diagnostic and prognostic ability in severely ill patients. The benefit of this knowledge we could gain for our future patients is obvious and high.

7. Scientific method

Search of medical databases using search engines (PUBMED, EMBASE, Web of Science, Cochrane Library for MESH Pancreatic stone protein, PSP, PSP/reg).
Statistic analysis of raw and coded data form eligible studies.

7.1 Statistical analysis

First metaanalysis – Survival:

Primary endpoint:
- Mortality

Analysis:
We will calculate sensitivity and specificity of PSP for the prediction of mortality due to sepsis, using mixed-effects models with the study included as a random effect. We will use all cut-off values reported

in the studies, namely 24, 130, and 177 ng/ml PSP. We will show sensitivity and specificity for every cut-off and every study, and a meta-analysed sensitivity and specificity.

In a next step, we anticipate a prognostic model for survival of patients, including PSP and optional further variables which are common and available in all studies as predictors in the model. We will split the available data into a test set and a validation set. In the validation set we will only include the newest publication, all other publications will be included in the test set. We will optimize the model using an internal 5-times 10-fold cross-validation on the test-set. The model will further be validated externally on the validation set.

Second metaanalysis – Diagnosis of sepsis:
Primary endpoint:

- Sepsis

Analysis:
We will calculate sensitivity and specificity of PSP for the diagnosis of sepsis, using mixed-effects models with the study included as a random effect. We will use all cut-off values of 30, 48.1, and 96.6 ng/ml PSP for the diagnosis of sepsis reported in the studies. We will show sensitivity and specificity for every cut-off and every study, and a meta-analysed sensitivity and specificity.

To test the value of PSP for the diagnosis of sepsis, we will try to find the best cut-off value. We will split the available data into a test set and a validation set. In the validation set we will only include the newest publication, all other publications will be included in the test set. We will first define a set of cut-off values, and then calculate sensitivity and specificity for each of these cut-off values. We will then define the optimal cut-off value, and internally and externally validate this cut-off value.

7.2 Design and outcome measures

Primary objectives:
To evaluate the role (to calculate the sensitivity and specificity) of PSP in:
- The diagnosis of sepsis/infection (4 studies)
- The prognosis of survival in sepsis (5studies)

Secondary objectives:
- The comparison with other known and current infection markers - CRP, PCT

8. Reporting obligation
NA

9. Data protection:
Coding and storage
We will receive coded individual patient data from the first or last authors of the original studies. We have no access to the code-list of these studies. For study (1), where the PI was first author, he is storing the code-list in a safe place.

Which persons are authorized for propagation of the health related data
The PI's of the original publications, i.e. Yok-Ai Que (1), Martin Llewelyn (2), Raphael Gukasjan (3), Marius Keel (4), Lukas Boeck (5), Luis García de Guadiana-Romulado (6), and Holger J. Klein (7).
Who takes responsibilty for the receipt of the data
We only receive data in coded form and have no access to the code list, and thus no access to un-coded patient data. Josef Prazak, MD is responsible for the coded data.

An exception is study (1), where the PI of the current study was involved as well. For data of this publication, he (Yok-Ai Que) will be responsible.

Who should have access to the health related personal data
We only receive data in coded form and have no access to the code list, and thus no access to the un-coded patient data. Following persons have access to the coded data:
Josef Prazak, MD
Yok-Ai Que MD, Prof
Armando Lenz, Statistician

Yok-Ai Que will further have access to the un-coded data of the publication he was first author (1).

Who is responsible for the protection of the data
The PI's of the original publications. We only have access to the coded data, without access to the code list, and thus no access to un-coded patient data. Josef Prazak, MD will be responsible for the protection of the coded data. Yok-Ai Que will further be responsible for the un-coded data of the publication he was first author (1).

10. Approach to uncoded data
NA

11. Declaration for data storage

Data will be coded and stored in a HFG conform database (audit-safe sharepoint server). The database format will be Excel/Sharepoint. This database can be accessed by the study team only.

12. Duration of storage
Data will be stored for 10 years in accordance with Swiss law.

13. Ethical and regulatory requirements
The project will be carried out in accordance to the protocol and with principles enunciated in the current version of the Declaration of Helsinki, the guidelines of Good Clinical Practice (GCP) issued by ICH, the Swiss Law and Swiss regulatory authority's requirements (HFG and HFV). This study was approved by the responsible ethics committee (Kantonale Ethikkommission Bern).

14. Funding / Publication / Conflict of interest
The study is partially funded by Abionic
Publication is planned to be submitted in a peer-review journal.
There is no conflict of interest by study team members.

15. Literature

1. Que, Y-A. et al, Prognostication of Mortality in Critically Ill Patients With Severe Infections, Chest, 2013
2. Llewelyn, M., et al., Sepsis biomarkers in unselected patients on admission to intensive or high-dependency care, Crit Care Med, 2013
3. Gukasjan, R., et al., Pancreatic Stone Protein Predicts Outcome in Patients With Peritonitis in the ICU, Crit Care Med, 2013
4. Keel, L., et al., Pancreatic stone protein is highly increased during posttraumatic sepsis and activates neutrophil granulocytes, Crit Care Med, 2009
5. Boeck, L., et al., Pancreatic Stone Protein A Marker of Organ Failure and Outcome in Ventilator-Associated Pneumonia, Chest, 2011
6. Guadiana-Romualdo, L.G., et al. Pancreatic stone protein and soluble CD25 for infection and sepsis in an emergency department, Eur J Clin Invest, 2017
7. Klein, H.J., et al., Pancreatic Stone Protein Predicts Postoperative Infection in Cardiac Surgery Patients Irrespective of Cardiopulmonary Bypass or Surgical Technique, PlosONE, 2015
