# Supplementary material for: Accuracy of pancreatic stone protein for the diagnosis of infection in hospitalized adults: a systematic review and individual patient level meta-analysis
Source: Crit Care. 2021 May 28;25:182. doi: 10.1186/s13054-021-03609-2 (PMC8164316; doi:10.1186/s13054-021-03609-2)

**Supplemental Figure 1: Distribution of PSP (A,B) [ng/ml], PCT (C,D) [ng/ml] and CRP (E,F) [mg/l] per study for patients with and without infection**.

**(A, C, E)** original y-axis. **(B,D,F)** y-axis adjusted for better visibility. Klein study did not assess PCT.


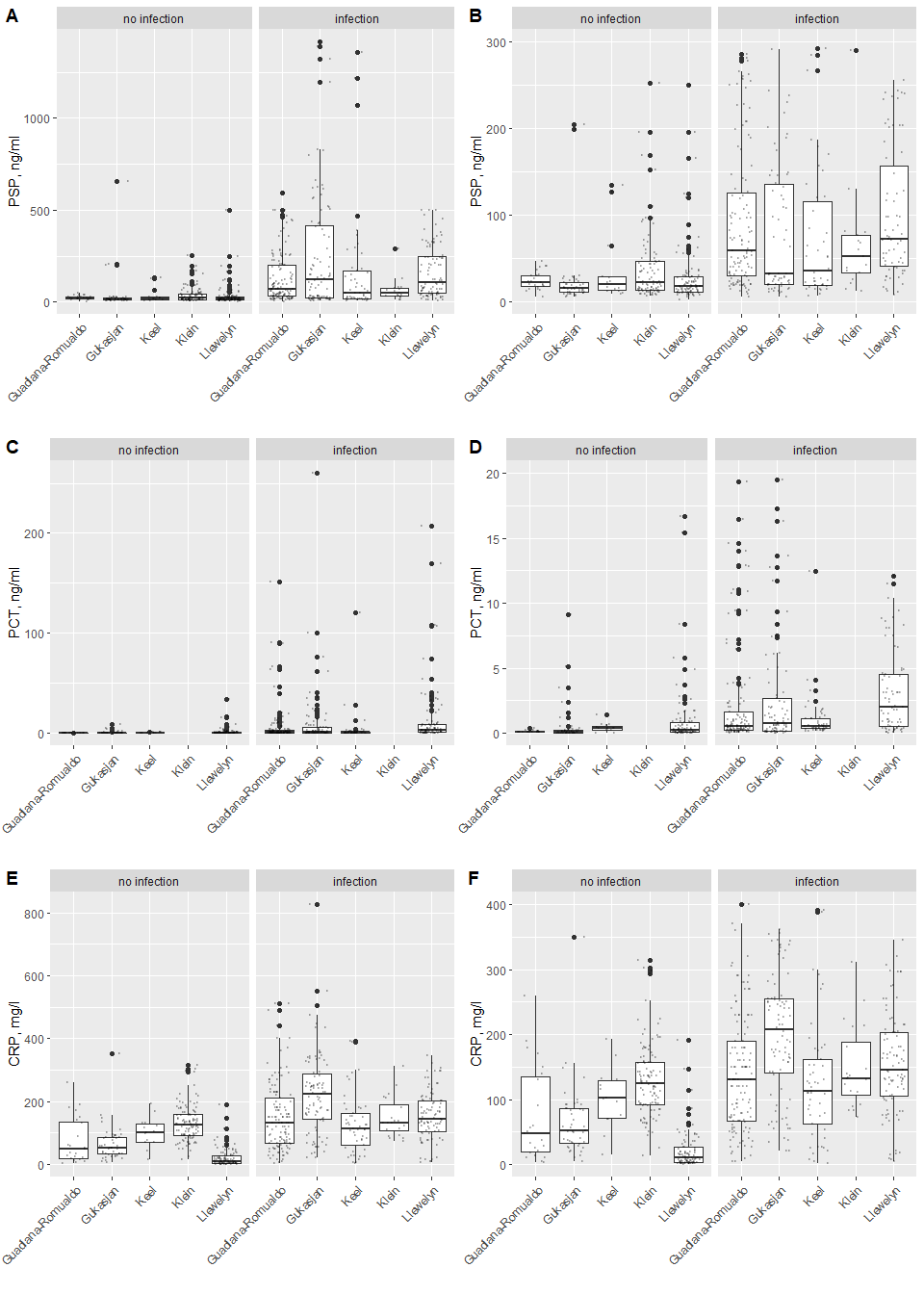


**Supplemental Figure 2**: **Log-transformed PSP, PCT, CRP.**

log PSP is non-linearly associated with log CRP and quite strongly linearly associated with log PCT, while not at all associated with log WBC. The log PSP distribution is tighter.


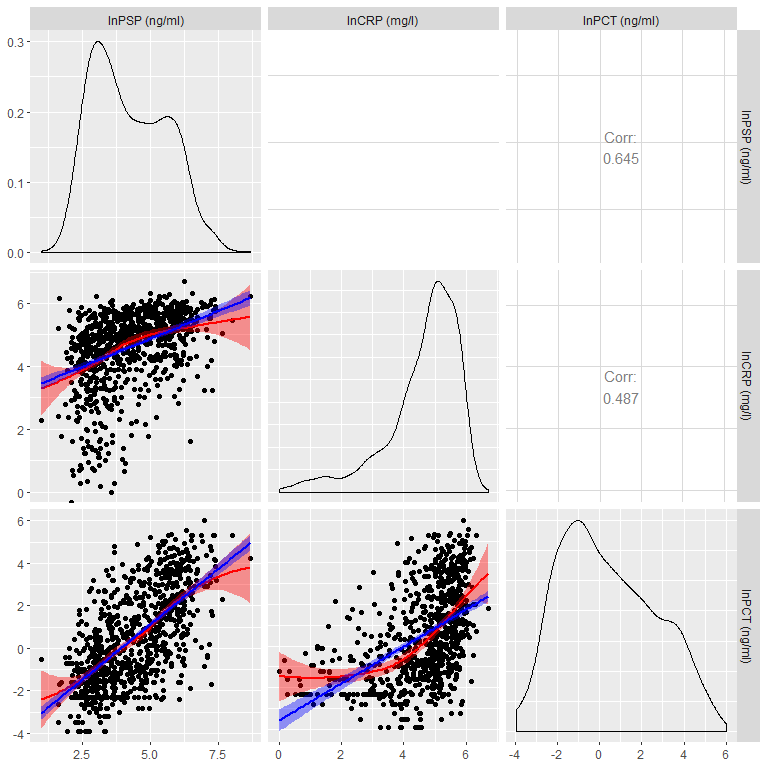


**Supplemental Figure 3 (3A and 3B): Distribution of PSP, CRP and PCT values**.

Values from infected patients are represented as red-circles; while values from non-infected patients are displayed as black circles crosses.

**Supplemental Figure 3A**: **Boxplot for untransformed PSP, CRP and PCT.**

Grey white-filled circles mark outliers (observations outside median +/- 1.5*interquartile range). The p-values for the null hypothesis ‘no difference in biomarker’ distribution for infection and no-infection’ are 7.05e-41, 1.21e-30 and 9.22e-26 for PSP, CRP and PCT respectively as computed with Kruskal-Wallis non-parametric test.


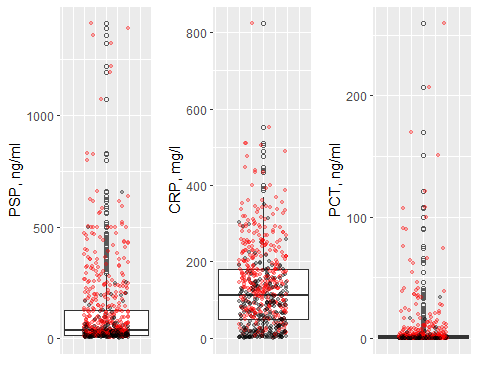


**Supplemental Figure 3B**: **Boxplot on the log2 scale for PSP, CRP and PCT.**

One CRP zero value and 16 zero PCT values were dropped for this visualization (not for subsequent analysis). Grey white-filled circles mark outliers (observations outside median +/- 1.5*interquartile range). The p-values for the null hypothesis ‘no difference in biomarker’ distribution for infection and no-infection’ are 7.05e-41, 1.21e-30 and 9.22e-26 for PSP, CRP and PCT respectively as computed with Kruskal-Wallis non-parametric test.


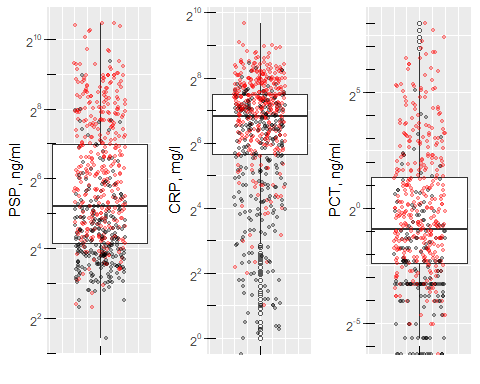


**Supplemental Figure 4**: **Forest plot showing estimated multivariable logistic regression coefficients and associated 95% confidence interval per study (with intercept and PSP effect) for predicting infection status**.

G-R stands for Guadiana-Romualdo et al.


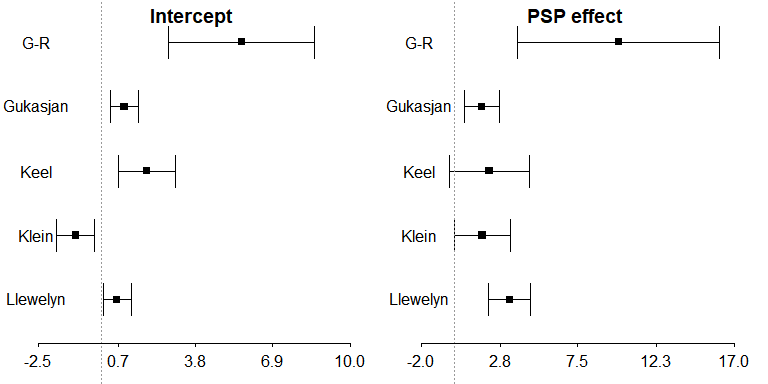


**Supplemental Figure 5: Funnel plots for Intercept and PSP effect for the fully stratified model.** The p-values for asymmetry are respectively 0.41 and 0.27.


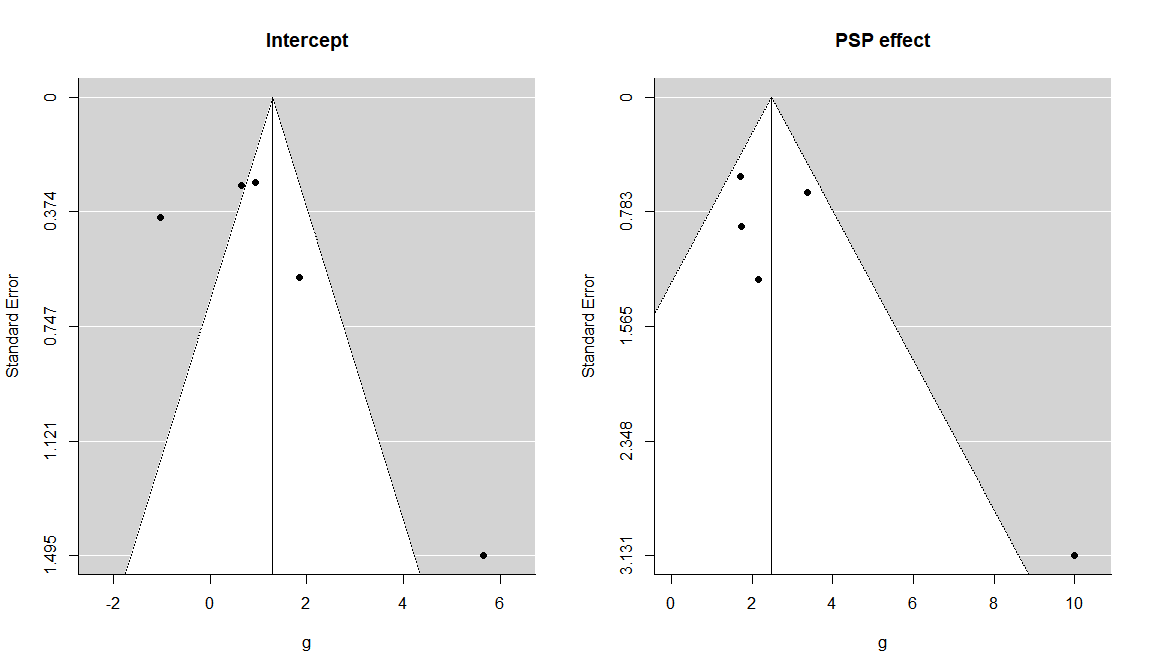


**Supplemental Figure 6: ROC curves computed using the predefined cutoffs as determined by the Youden’s Index (see Table 4) on each of the individual datasets of the five studies**.

**(A)** PSP [cut off 44.18 ng/l]; **(B)** CRP [99.50 mg/l]; **(C)** PCT [0.20 ng/ml]. Klein et al. did not report any PCT values.


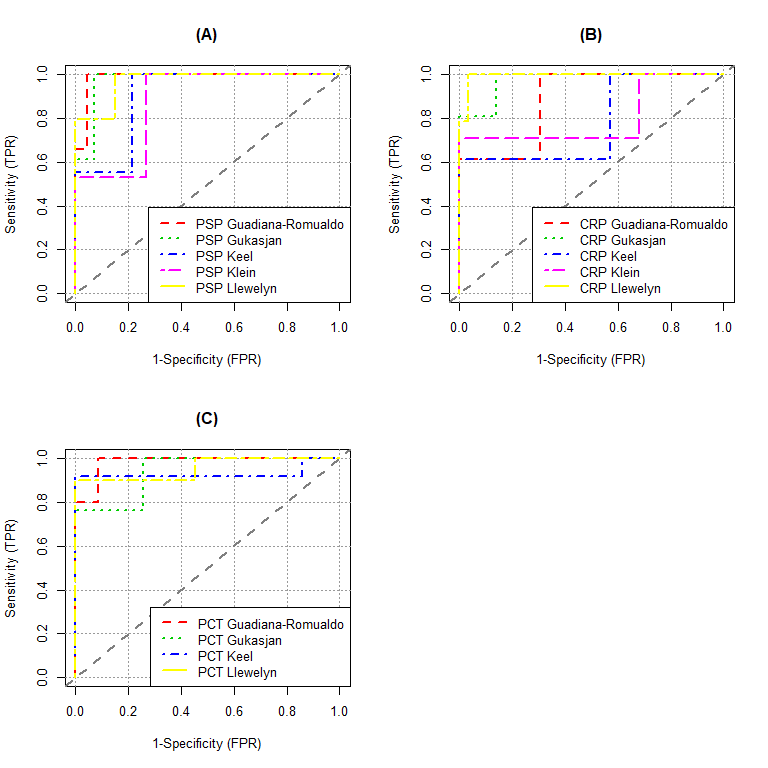


**Supplemental Figure 7: Predicting infection versus non-infection status using joint models**.

**(A)** PSP+CRP leading to an AUC of 0.90 (95%CI, 0.87, 0.92); **(B)** PSP+PCT leading to an AUC of 0.83 (0.80, 0.87) and PSP+CRP+PCT to an AUC of 0.90 (0.87, 0.92). PSP+CRP+PCT ROC curve completely coincides with PSP+CRP ROC curve, therefore by parsimony principle the PSP+CRP model was preferred.


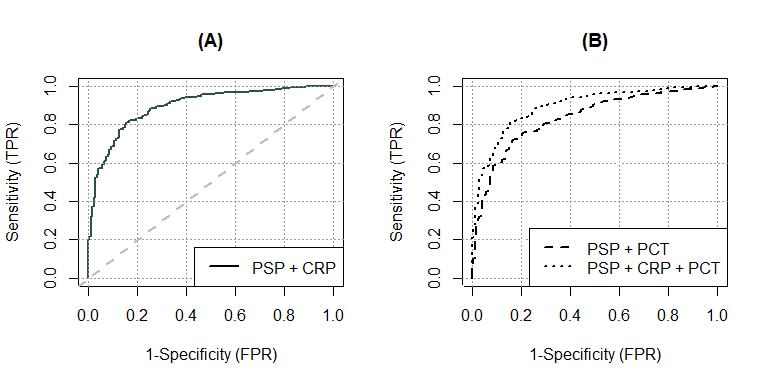

Supplement: Supplementary file 3 — Additional file 3. Supplemental Figures. [file 13054_2021_3609_MOESM3_ESM.doc]
