# Supplementary material for: Accuracy of pancreatic stone protein for the diagnosis of infection in hospitalized adults: a systematic review and individual patient level meta-analysis
Source: Crit Care. 2021 May 28;25:182. doi: 10.1186/s13054-021-03609-2 (PMC8164316; doi:10.1186/s13054-021-03609-2)
Supplement: Supplementary file 4 — Additional file 4. Supplemental Methods. [file 13054_2021_3609_MOESM4_ESM.docx]

**Supplemental Material**

**SUPPLEMENTAL METHODS**

To select the meta-analysis model with the appropriate degree of heterogeneity in baseline risk and predictor effect, we followed the approach proposed by Steyerberg *et al*. (12) and evaluated the following three models (Supplemental Table 4 and 5).

## ***‘Fully stratified’ random-effect model.***

The infection status of subject $i$ from study $j$ follows a Bernoulli distribution:

$${infection\_status}_{ij}\sim\mathrm{Bernoulli}(p_{ij})$$

$$\mathrm{logit} p_{ij}=\alpha_{j}+\beta_{j} \mathrm{PSP}_{ij}$$

$$\alpha_{j},\beta_{j}\sim MVN(\mu,T).$$

The model is fit in two stages. First, a logistic regression model is fitted in every study separately, yielding study-specific estimates ${\overset{^}{\alpha}}_{j}$ and ${\overset{^}{\beta}}_{j}$ (Supplemental Figure 2, Supplemental Table 5A and Supplemental Table 6). The individual patient data allows the computation of $S$- the full within-study covariance matrix of the estimates ${\overset{^}{\alpha}}_{j}$ and ${\overset{^}{\beta}}_{j}$. The parameters of the global prediction model are estimated from the model

$\alpha_{j},\beta_{j}\sim MVN(\mu,T+S)$.

In these notations, $T$ is the between-study covariance matrix of the pooled intercept and predictor effect. Compared to the two-stage approach, the one-stage approach would require considerable computational effort to provide similar results (2-4).

## ***Mixed-effect model***

In the mixed-effect model, we assume that the PSP effect between studies is common, but the intercepts may vary between studies reflecting a different baseline risk of infection.

$$\mathrm{logit} p_{ij}=\alpha_{j}+\beta\mathrm{PSP}_{ij}$$

$$\alpha_{j}\sim N(\mu_{\alpha},\tau_{\alpha}^{2}).$$

We fit this model as mixed-effect logistic regression model, the results in terms of ROC AUC are presented in Supplemental Tables 5B and 7.

## ***Fixed-effect model***

In the fixed-effect model, we assume that both the intercept and PSP effect are common between all the considered studies:

$$\mathrm{logit} p_{ij}=\alpha+\beta\mathrm{PSP}_{ij}.$$

The respective estimates and ROC AUC for PSP data are in Supplemental Tables 5C and 9.

**REFERENCES**

1. Steyerberg EW, Nieboer D, Debray TPA, van Houwelingen HC. Assessment of heterogeneity in an individual participant data meta-analysis of prediction models: An overview and illustration. *Statistics in medicine* 2019; 38: 4290-4309.
2. Debray, T.P., Moons, K.G., Ahmed, I., Koffijberg, H. and Riley, R.D. A framework for developing, implementing, and evaluating clinical prediction models in an individual participant data meta‐analysis. Statist. Med. 2013; 32:3158-3180.
3. Debray, T. P., Schuit, E., Efthimiou, O., Reitsma, J. B., Ioannidis, J. P., Salanti, G., Moons, K. G. M. on behalf of GetReal Workpackage (2016). An overview of methods for network meta-analysis using individual participant data: when do benefits arise? Statistical Methods in Medical Research, 27(5), 1351–1364.
4. Ewout W. Steyerberg, Daan Nieboer, Thomas P.A. Debray, Hans C. Houwelingen, Assessment of heterogeneity in an individual participant data meta‐analysis of prediction models: An overview and illustration, Statistics in Medicine. 2019; 38: 4290-4309.
