# Supplementary material for: Accuracy of pancreatic stone protein for the diagnosis of infection in hospitalized adults: a systematic review and individual patient level meta-analysis
Source: Crit Care. 2021 May 28;25:182. doi: 10.1186/s13054-021-03609-2 (PMC8164316; doi:10.1186/s13054-021-03609-2)
Supplement: Supplementary file 5 — Additional file 5. Supplemental Material. [file 13054_2021_3609_MOESM5_ESM.doc]

**SUPPLEMENTAL MATERIAL – Gukasjan’S PUBLICATION CONTROL GROUP**

We received data from 46 additional patients recruited from 23.07.2007 to 16.06.2009 together with the patients presented in Gukasjan’s original publication. The study protocol as well as the definition of this unpublished control group were described in the NCT01465711 study file. Three patients’ records had incomplete data and were excluded from the meta- analysis, leaving 43 additional patients with uneventful course after elective major abdominal surgery.

**Supplemental Table:** Characteristics of the 43 additional patients admitted electively to the ICU after uneventful elective major abdominal surgery.

| Publication and infection status | Age | Men  (%) | PSP ng/ml [IQR] | CRP mg/l  [IQR] | PCT ng/ml  [IQR] |  |
| --- | --- | --- | --- | --- | --- | --- |
|  |  |  |  |  |  |  |
| Gukasjan et al.  Patient without infection  n=43 | 55 [50, 65] | 21 (49% ) | 15.22 [11.21, 23.24] | 51.3 [32.7, 86.4] | 0.1 [0.03, 0.2] |  |
